# The great debate at “Melanoma Bridge 2018”, Naples, December 1st, 2018

**DOI:** 10.1186/s12967-019-1892-5

**Published:** 2019-05-10

**Authors:** Paolo A. Ascierto, Paolo Bruzzi, Alexander Eggermont, Omid Hamid, Hussein A. Tawbi, Alexander van Akkooi, Alessandro Testori, Corrado Caracò, Igor Puzanov, Francesco Perrone

**Affiliations:** 1 Unit of Melanoma, Cancer Immunotherapy and Innovative Therapy, Istituto Nazionale Tumori IRCCS Fondazione “G. Pascale”, Via Mariano Semmola, 80131 Naples, Italy; 2 Clinical Epidemiology Unit, University Hospital “San Martino”, Genoa, Italy; 3 Department Melanoma, Soft Tissue, Muscle-Skeletal and Head-Neck, Istituto Nazionale Tumori IRCCS Fondazione “G. Pascale”, Naples, Italy; 4 Institut Gustave Roussy Villejuif, Paris-Sud, VilleJuif, Paris, France; 5 Clinical Research and Immunotherapy, The Angeles Clinic and Research Institute, Los Angeles, CA USA; 6 Department of Medicine, Roswell Park Comprehensive Cancer Center, Buffalo, NY USA; 7 Melanoma Clinical Research & Early Drug Development, Melanoma Medical Oncology, Investigational Cancer Therapeutics, UT MD Anderson Cancer Center, Houston, TX USA; 8 Surgical Group, EORTC Melanoma Group, Brussels, Belgium; 9 Department of Surgical Oncology, Netherlands Cancer Institute – Antoni van Leeuwenhoek Hospital (NKI-AVL), Amsterdam, The Netherlands; 10 Clinical Trials Unit, Istituto Nazionale Tumori IRCCS Fondazione “G. Pascale”, Naples, Italy

**Keywords:** Melanoma, Immunotherapy, Anti-PD-1, Anti-CTLA-4, Targeted therapy, BRAF inhibitor, MEK inhibitor, Adjuvant, Neoadjuvant

## Abstract

The great debate session at the 2018 Melanoma Bridge congress (November 29–December 1, Naples, Italy) featured counterpoint views from experts on three topical issues in melanoma. These were whether overall survival should still be the main endpoint for clinical trials in melanoma, whether anti-cytotoxic T-lymphocyte-associated antigen (CTLA)-4 is still the optimal choice of drug to use in combination with an anti-programmed death (PD)/PD-ligand (L)-1 agent, and the place of adjuvant versus neoadjuvant therapy in patients with melanoma. These three important debates are summarised in this report.

## Introduction

The great debate session at the 2018 Melanoma Bridge congress (November 29–December 1, Naples, Italy) featured counterpoint views from experts on three topical issues in melanoma. These were whether overall survival (OS) is still the best endpoint for clinical trials in melanoma, whether anti-CTLA-4 is still the optimal choice of drug to combine with an anti-PD-L1 agent, and the place of adjuvant versus neoadjuvant therapy in patients with melanoma. These three important debates are summarised in this report.

## Is overall survival still the main endpoint? Yes or no

### Alexander Eggermont: yes

Selective kinase inhibitors and immune checkpoint blockers have both significantly prolonged survival of patients with advanced metastatic melanoma. Combined BRAF and MEK inhibition has an immediate measurable effect in patients with metastatic disease with few relapses until 9 months. With anti-PD-1s, around 20% of patients do not respond. Survival curves for anti-PD-1 based regimens (monotherapy or combined with ipilimumab) and combined BRAF inhibitor plus MEK inhibitor cross at around 12–16 months so that anti-PD-1-based therapy has a superior survival benefit onwards [1]. Ipilimumab alone is associated with stable long-term survival in around 20% of patients.

In the European Organisation for Research and Treatment of Cancer (EORTC) 18071 trial of adjuvant ipilimumab after complete resection of stage III cutaneous melanoma, 5-year rate of recurrence-free survival (RFS) at a median follow-up of 5.3 years was 41% with ipilimumab compared to 30% with placebo (hazard ratio [HR] for recurrence or death, 0.76; P < 0.001) [2]. This 11% absolute difference in RFS is maintained in overall survival (OS), with 5-year OS rates of 65% in the ipilimumab group versus 54% in the placebo group (HR for death, 0.72; P = 0.001). Because the placebo group were not systematically rescued after relapse, there was very little crossover between arms, with only 23% of placebo-treated patients receiving immunotherapy after relapse. OS after disease recurrence was similar in the two treatment groups (HR 0.89), suggesting that benefits gained were due to the adjuvant phase and that the treatment difference in RFS would persist in terms of OS. The rate of distant metastasis-free survival (DMFS) at 5 years was also consistent with RFS and OS (48% with ipilimumab and 39% with placebo, HR for death or distant metastasis, 0.76; P = 0.002). However, treatment was associated with very problematic toxicity, with grade 3–4 adverse events occurring in 54% of patients in the ipilimumab group and five patients dying due to immune-related adverse events.

In the CheckMate 238 trial of patents undergoing resection of stage IIIB/C-IV melanoma, adjuvant treatment with nivolumab resulted in a 1-year RFS rate of 71% versus 61% with ipilimumab 10 mg/kg (HR for disease recurrence or death, 0.65; P < 0.001) [3]. At 2 years, RFS continued to be significantly longer with nivolumab versus ipilimumab with a 13% absolute difference (63% versus 50%) [4]. Two-year RFS rates were higher for nivolumab than ipilimumab for subgroups defined by disease stage, PD-L1 expression and BRAF mutation status. DMFS was also significantly better with nivolumab although by a slightly lesser magnitude (HR 0.76, P = 0.034).

Adjuvant pembrolizumab was also associated with significantly longer RFS versus placebo in the KEYNOTE-054 trial, with results consistent with those seen with nivolumab (1-year RFS rate, 75% vs. 61%; HR for recurrence or death, 0.57; P < 0.001) [5]. There was no significant difference in RFS by PD-L1 expression or BRAF status. DMFS was consistent with RFS. Toxicity was low, with grade 3–5 adverse events reported in 14.7% of patients in the pembrolizumab group, and less than observed in the COMBI-AD trial of combined BRAF/MEK inhibition.

In COMBI-AD, dabrafenib plus trametinib resulted in 3-year rate of RFS of 58% in the combination group and 39% in the placebo group (HR for relapse or death, 0.47; P < 0.001) [6]. Improved RFS also translated into improved DMFS and OS. However, around one-quarter of patients stopped treatment because of toxicity, indicating that PD-1-based treatment is the best tolerated. Checkpoint inhibitor therapy also offers better survival rates than dabrafenib plus trametinib after 3–4 years. Immune gene expression signatures (e.g. interferon [IFN]-γ signature) were strongly prognostic for RFS in COMBI-AD [7]. IFN-γ gene signature identified patients with longer RFS independently of tumor mutational burden in the combination therapy group.

Whether adjuvant therapy is necessary for improved OS may be a question for debate. Standard-dose pembrolizumab in combination with reduced-dose ipilimumab resulted in 12-month OS of 89% in the KEYNOTE-029 trial of patients with advanced melanoma [8]. Given such high OS, adjuvant therapy may not be worthwhile.

Because of the cross-over design, only the adjuvant pembrolizumab trial formally addresses the question of whether adjuvant pembrolizumab immediately after surgery in all patients provides a benefit over treatment with pembrolizumab starting at the time of relapse. It is good that at least one trial formally addresses this question in spite of the overall observation in a meta-analysis that the impact on RFS generally correlates well with an impact on OS [9].

These new therapies mean adjuvant IFN-α will no longer be used, other than for patients with ulcerated melanoma in countries without access to other treatment options [10].

In the future, it will be important to better understand the duration of treatment that is required, e.g. 1 versus 2 years of dabrafenib plus trametinib. New immunotherapy combinations need to be explored, such as nivolumab with low-dose ipilimumab, talimogene laherperepvec (T-Vec) or toll-like receptor (TLR)-9 agonists. Neoadjuvant approaches will also be important.

### Paolo Bruzzi: no

Clear definitions are needed in this discussion because treatment aims, trial aims and trial endpoints are not necessarily the same. Treatment aims are generally for the patient to live longer, and/or with an improved quality of life, and any expectation focused on intermediate aims (e.g. improved disease control, tumor shrinkage, delayed progression) is conditional on the assumption that this will translate into a benefit in survival or quality of life. The aim of a trial is to prove or disprove that the study drug is effective in producing the effects searched by the patient, that is, in that it prolongs survival or improves quality of life, while the trial endpoints are the measurements used to achieve the aims of the trial. As a consequence, OS and quality of life scores appear as the ‘natural’ endpoints in cancer trials.

Clinical trials frequently use surrogate endpoints, which are clinical, laboratory or instrumental variables that can be used as the primary endpoint and which allow the effects of treatment on the natural endpoint (i.e. survival) to be estimated. Surrogate endpoints are convenient because they allow a shorter time to interim and final analysis and also typically provide a stronger treatment effect. For example, in advanced cancers, time to a progression-free survival (PFS) endpoint is approximately one-third of the time taken to obtain an OS result. A meta-epidemiological study reported that trials that report surrogate primary outcomes are also more likely to report larger treatment effects than trials reporting final patient-relevant primary outcomes [11]. This finding was not explained by differences in the risk of bias or characteristics of the two groups of trials. This phenomenon, termed dilution, has a simple arithmetical explanation and implies that many less patients are needed to demonstrate the presence of an effect on a surrogate endpoint such as response rate or PFS than an effect on survival.

As a consequence, if OS is used as the main endpoint in a clinical trial the time required for obtaining the final results may be too long and, most importantly, clinically significant treatment effects can be overlooked. Because of this, the US Food and Drug Administration (FDA) has become much more flexible with regard to providing accelerated approval of new drugs based on surrogate endpoints that are reasonably expected to predict clinical benefit. However, there are disadvantages in the use of surrogate endpoints. First, overall response rate (ORR), PFS and RFS are all subject to assessment bias and this can never be entirely ruled out although the bias can be mitigated if responses/progressions are assessed in a blinded fashion. Second, there are examples of false negative results, that is trials in which, despite the lack of a clear effect on a surrogate endpoint, an effect on OS was present, and this problem seems to be typical of immunotherapy trials. Third, and more worrisome, false positive effects can occur with subsequent overestimation of the benefit of a new drug. False positive results can occur due to dilution, with PFS but not OS statistically significant, but in these cases the surrogate may still be a valid surrogate. The surrogate endpoint can also still be valid in cases of crossover of treatment after progression, that result in a non-significant difference in OS despite a significant PFS difference. However, in other situations (e.g. biological effects such as clonal selection), a surrogate endpoint may be truly invalid, and its use as the primary endpoint in a clinical trial may provide misleading indications on the efficacy of the experimental treatment.

It is important to note that the validity of a surrogate endpoint is disease-, drug- and endpoint-specific. Validation of a surrogate endpoint is challenging. Approaches include Prentice’s four criteria [12], although this requires a very large original database from randomized controlled trial(s) showing the drug effect on the true endpoint with the demonstration that OS depends entirely on the surrogate endpoint and not on the drug. This is cumbersome and seldom used, although an example of this is the demonstration from the FDA that showed a strong association between ORR and PFS in patients with advanced non-small cell lung cancer (NSCLC) treated with mainly targeted therapies [13]. Another approach is that of Buyse et al. [14] which involves meta-analytic (trial level) validation. However, this requires several trials that can be pooled to show a correlation. An example of this is a meta-analysis of 12 randomized trials in metastatic melanoma that noted a strong correlation between the treatment effects for PFS and OS [15]. This concluded that PFS can be regarded as a robust surrogate for OS in dacarbazine-controlled randomized trials of metastatic melanoma. One problem with this approach though is if no effect on PFS is observed.

Given the consideration that the requirements for both these approaches is very stringent and challenging, with the need for large databases and/or several clinical trials, another approach to validation is needed. What we need to assess is post-progression survival. PFS and OS should be co-primary trial endpoints (with multiplicity correction). If no significant difference is observed in PFS, the OS results need to be awaited. However, if there is a significant difference in PFS, a new testable null hypothesis arises, which is whether the absolute benefit in PFS translates into a similar increase in OS. This is equivalent to assuming that the post-progression survival is the same in both arms. Unless significant differences in post-progression survival are observed, it can be concluded that the null hypothesis is true i.e. that the benefit in PFS is maintained and translates into the same benefit in OS. This is true irrespective of whether the OS difference is statistically significant or not. If post-progression survival does differ, this needs to be investigated to assess why (e.g. can it be due to crossover or was the frequency of crossover insufficient to justify it?). Validation of surrogate endpoints is a priority that should be important to clinical researchers as well as statisticians.

In conclusion, OS may still be the main endpoint, but hopefully this will not always remain the case.

### Key points

**Fig. 1:**
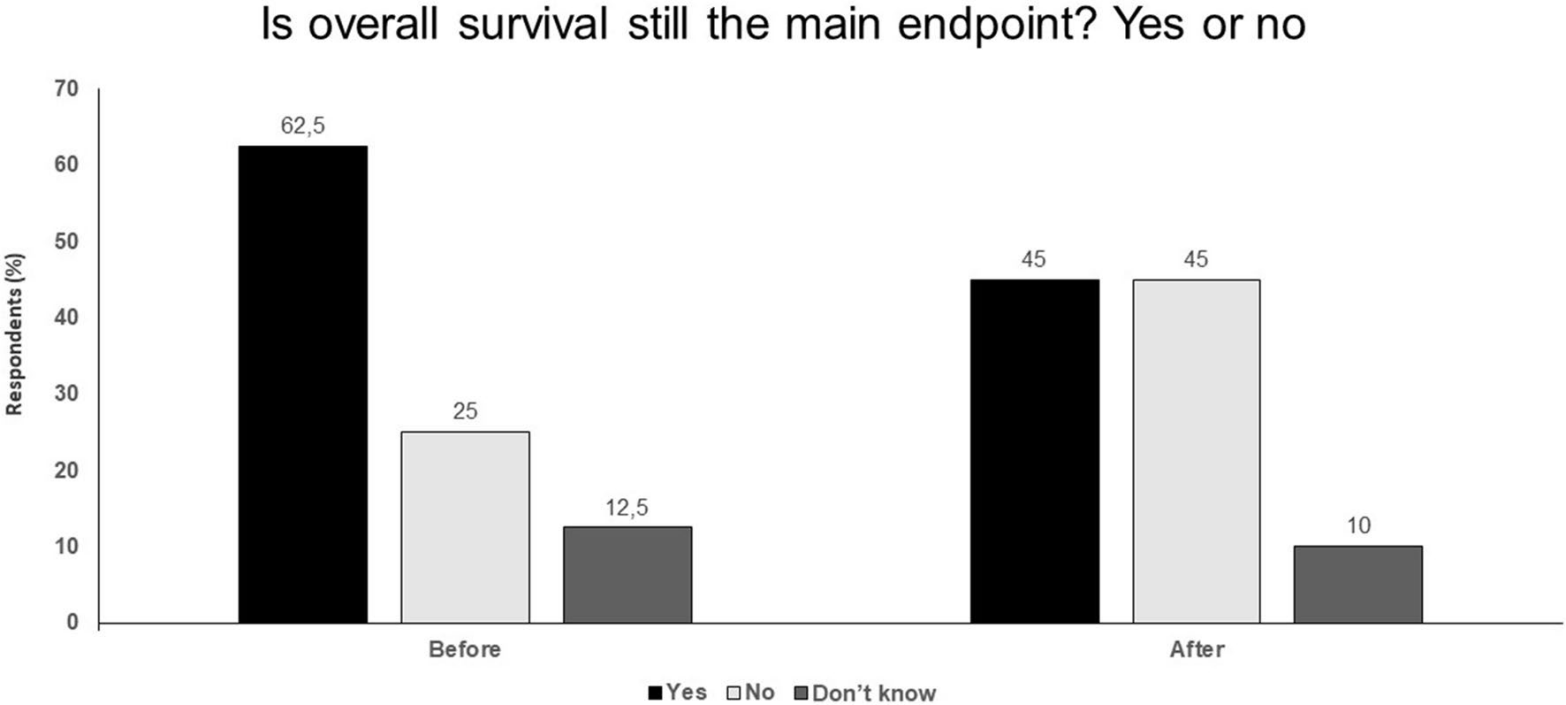
Is overall survival still the main endpoint? Yes or no. Audience response before and after debate

- Efficacy of treatments in advanced melanoma translates directly into benefits in the adjuvant setting.
- In BRAF-mutant melanoma, adjuvant therapy with BRAF plus MEK inhibitors provides the best initial benefit, but from around 2 years and beyond, anti-PD-1 therapies seem to have better results.
- Anti-PD-1 plus anti-CTLA-4 in advanced melanoma has such impressive results that the impact of adjuvant therapies on OS still remains to be proven.
- Clinical trials frequently use surrogate endpoints which can be a convenient means to allow the effects of treatment on the natural endpoint (i.e. survival) to be estimated.
- If OS is used as the main endpoint, the time required for obtaining the final results may be too long and clinically significant treatment effects can be overlooked.
- Validation of surrogate endpoints should be a priority (Fig. 1).

## Is anti CTLA-4 still the optimal drug to combine with anti PD-L1? Yes or no

### Paolo A. Ascierto: yes

The CheckMate-067 trial in patients with previously untreated, unresectable stage III–IV melanoma showed that a durable survival benefit can be achieved with nivolumab plus ipilimumab or nivolumab alone, with 4-year survival rates of 53% and 46%, respectively [16]. These data appear to indicate that addition of ipilimumab to nivolumab does not offer a major clinical benefit. Moreover, assessment of post-progression therapy suggests that sequential use of ipilimumab after nivolumab can be beneficial, so that combination treatment may not be necessary.

However, PFS landmark analysis of the most important studies in advanced melanoma clearly indicate the advantage of combined anti-PD-1 and anti-CTLA-4 therapy and shows the importance of an early response with the combination. Other studies have also shown the benefit of combined nivolumab plus ipilimumab in different patient subgroups, including patients with brain metastases [17]. Other important evidence for the benefits of combined treatment is from neoadjuvant studies, where adding ipilimumab to anti-PD-1 almost doubled the complete pathological response rate (pCR) from 25% with nivolumab alone to 45% with combined nivolumab plus ipilimumab [18], which is likely to predict improved RFS. The subsequent OPACIN-NEO neoadjuvant trial reported a similar response rate with a different dose regimen of ipilimumab 1 mg/kg plus nivolumab 3 mg/kg but with more manageable toxicity [19]. Pathological response was correlated with RFS and a baseline IFN-α signature was identified as a possible biomarker for treatment outcome.

**Fig. 2:**
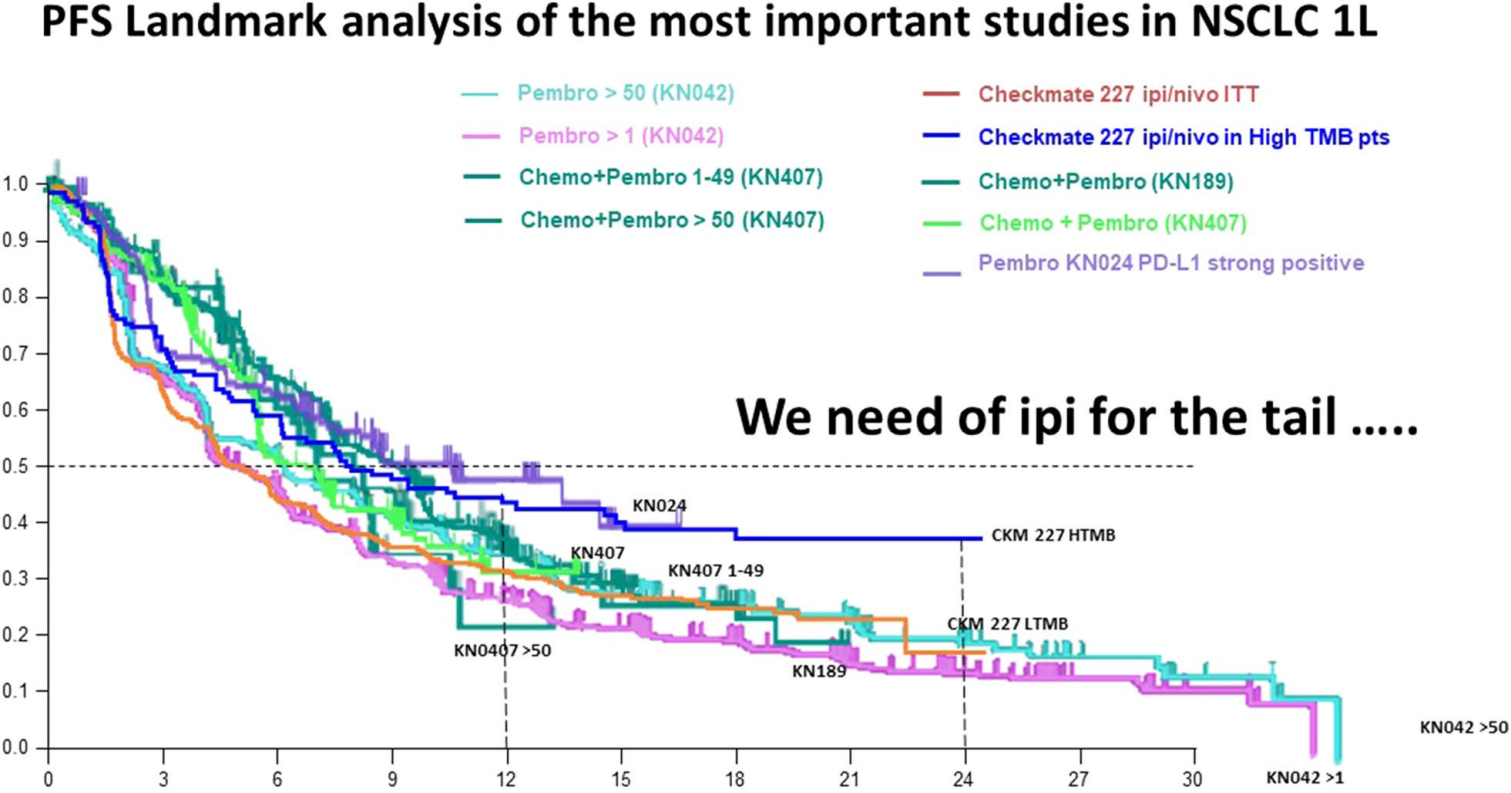
PFS landmark analysis of the most important studies in NSCLC 1L. Progression-free survival landmark analysis of the most important studies in non-small-cell lung cancer 1L

Another important consideration is related to the tail of the survival curve. In the CheckMate-067 trial, the PFS curve from 3 years onwards looks slightly better with combined nivolumab plus ipilimumab versus nivolumab alone [16]. Also, looking to the OS curves of pembrolizumab at 4 and 5 years in the KEYNOTE-006 and KEYNOTE-001 studies, these do not appear to be as flat as the anti-CTLA-4 OS curve [20, 21]. These findings appear to suggest that the memory effect is probably more important with ipilimumab. The addition of ipilimumab to nivolumab also appears to have a greater effect in patients with BRAF-mutated versus BRAF wild-type melanoma, and has also been shown to be effective in tumors other than melanoma, including urothelial cancer [22], gastric cancer [23], small-cell lung cancer [24] and NSCLC with a high tumor mutational burden [25]. As in advanced melanoma, PFS landmark analysis of the most important studies in first-line NSCLC also suggests that the addition of ipilimumab to anti-PD-1 therapy is beneficial for long-term durable response (Fig. 2).

It is likely that a higher dosage of ipilimumab offers more benefit; however, toxicity is a problem especially when given in combination. Ongoing clinical trials are investigating approaches to help increase ipilimumab activity or reduce toxicity. Ipilimumab-NF includes a non-fucosylated glycan which enhances antibody-dependent cellular cytotoxicity (ADCC) activity and increases T regulatory cell (Treg) depletion at the tumor and has the potential for reduced dosing, while ipilimumab-Probody™ has a masking peptide cleavable by tumor selective protease activity at the tumor site. This localizes anti-CTLA-4 activity to the tumor thereby reducing systemic CTLA-4 blockade and toxicity and offers the potential for higher dosing [26]. These developments may further increase the likelihood of a future role of anti-CTLA-4 in combination with anti-PD-1 therapy.

### Omid Hamid: no

Ipilimumab is a very potent and effective drug. If we are going to relegate it to being a watered down ‘second banana’ to PD-1 therapy. Then it should be used alone or in other combinations rather than in combination with PD-1. Ipilimumab 3 mg/kg has been shown to be effective in patients with brain metastases and patients with BRAF-mutated tumors and a memory effect with lower dose ipilimumab has also been demonstrated [16]. In advanced melanoma, ipilimumab at a higher dose of 10 mg/kg resulted in significantly prolonged OS rate compared with lower dose ipilimumab 3 mg/kg [27]. Given these considerations, why is the trend towards using ipilimumab at a reduced dose in combination with nivolumab, rather than as a more potent, higher-dose monotherapy?

In the CheckMate-511 study, the approved dose of nivolumab 1 mg/kg plus ipilimumab 3 mg/kg, as used in the CheckMate 067 trial, was compared to nivolumab 3 mg/kg plus ipilimumab 1 mg/kg in patients with advanced melanoma [28]. Treatment-related grade 3–5 adverse events were significantly lower in the nivolumab 3 mg/kg plus ipilimumab 1 mg/kg group. However, response rate numerically favored the nivolumab 1 mg/kg plus higher dose ipilimumab 3 mg/kg arm, although this was not significantly different. This study was not powered to choose a best regimen and should not be presented as so.

Nivolumab and ipilimumab have also been evaluated in combination in other solid tumors and have been associated with durable responses and long-term OS in heavily pretreated patients with advanced gastric, esophageal or gastroesophageal junction cancer [29]. Nivolumab 1 mg/kg plus ipilimumab 3 mg/kg looked to have slightly better PFS than nivolumab 3 mg/kg plus ipilimumab 1 mg/kg or nivolumab alone. However, differences between groups were minor, especially between the group receiving nivolumab monotherapy and with the addition of ipilimumab 1 mg/kg, with OS data suggesting no benefit from adding lower dose ipilimumab. The CheckMate-016 trial in patients with metastatic renal cell carcinoma has also suggested a greater survival benefit at 2 years with nivolumab 1 mg/kg plus ipilimumab 3 mg/kg versus nivolumab 3 mg/kg [30]. All these data suggest that ipilimumab may offer greater potential benefit if used at a higher dose; however, the general trend is towards utilizing in combination with anti-PD-1 treatment at a reduced dose to avoid toxicity problems. This may be a false path forward.

A better approach may be to reserve ipilimumab, alone or in combination with other agents, for patients who fail anti-PD-1 therapy. For example, ipilimumab in combination with the TLR9 agonist tilsotolimod (IMO-2125) has demonstrated substantial clinical benefit including durable responses in patients with PD-1 inhibitor refractory metastatic melanoma [31]. Other studies have also suggested that ipilimumab may be effective in patients who have progressed on anti-PD-1 treatment [32, 33]. If ipilimumab is used at a low dose in combination with nivolumab, this rescue option may not be available and the potential of CTLA-4 blockade is largely wasted with ipilimumab being done a disservice. New developments in CTLA-4 blockade, such as ipilimumab-NF and ipilimumab-Probody™, that aim to increase potency or reduce toxicity offer the potential for the use of higher doses or more frequent dosing as monotherapy or combination therapy.

### Key points

**Fig. 3:**
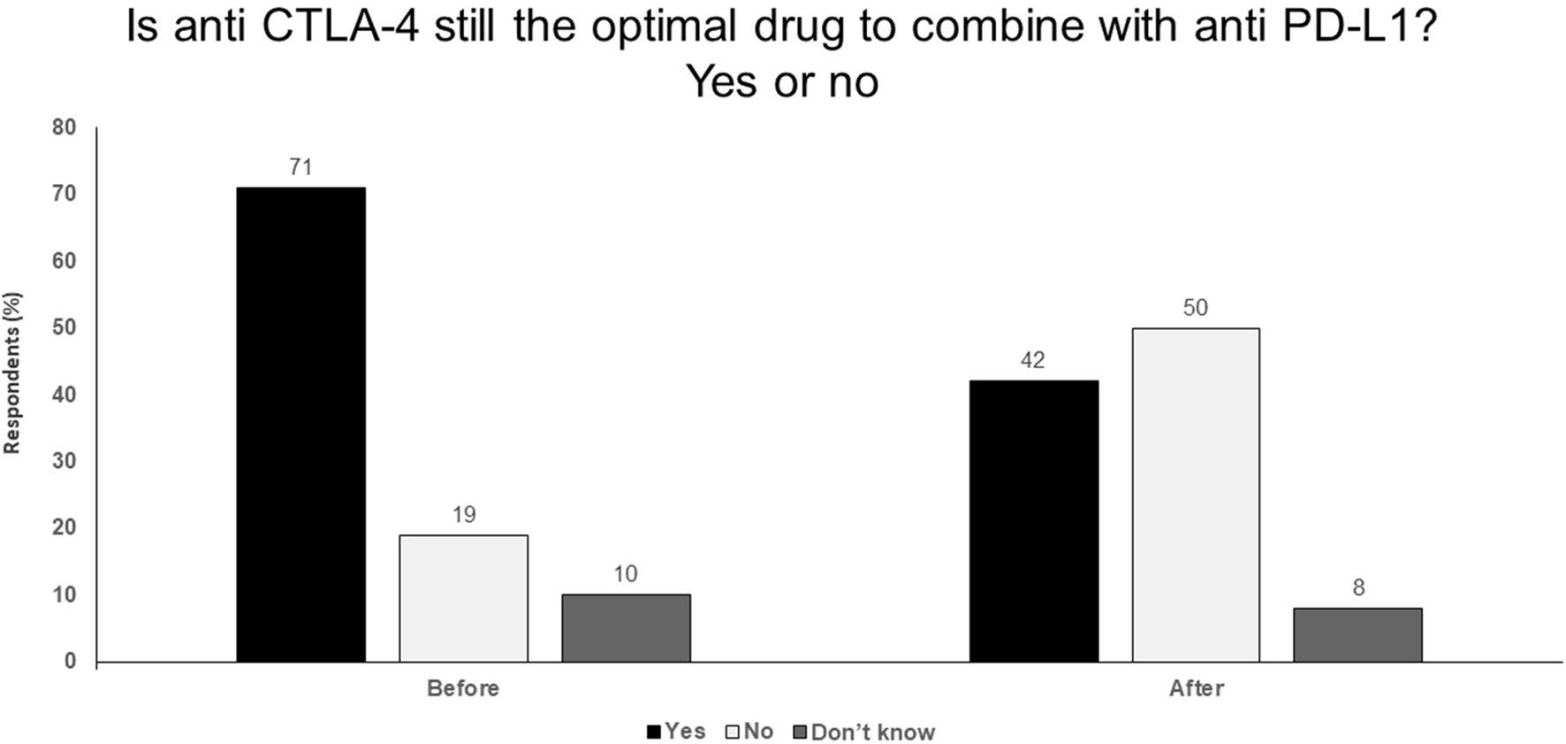
Is anti CTLA-4 still the optimal drug to combine with anti PD-L1? Yes or no. Audience response before and after debate

- PFS landmark analysis of important studies in advanced melanoma clearly indicate the advantage of combined anti-PD-1 and anti-CTLA-4 therapy and shows the importance of an early response with the combination.
- Another important consideration is related to the tail of the survival curve with the PFS curve from 3 years onwards looking move favourable with combined nivolumab plus ipilimumab versus nivolumab alone, suggesting that the memory effect may be more important with anti-CTLA-4 therapy.
- A higher dosage of ipilimumab offers more benefit but toxicity is a problem, especially in combination, so novel approaches to help increase activity or reduce toxicity are being investigated e.g. ipilimumab-NF, ipilimumab-Probody™.
- Ipilimumab is a very potent and effective drug.
- In advanced melanoma, ipilimumab at a higher dose of 10 mg/kg resulted in significantly prolonged OS rate compared with lower dose ipilimumab 3 mg/kg.
- New developments in CTLA-4 blockade, such as ipilimumab-NF and ipilimumab-Probody™, that aim to increase potency and reduce toxicity offer the potential for the use of higher doses or more frequent dosing as monotherapy or combination therapy (Fig. 3).

## Adjuvant versus neoadjuvant therapy

### Alexander van Akkooi: in favor of adjuvant

There is strong evidence from prospective, phase III randomized controlled trials that adjuvant therapy is effective. In a phase III trial in patients who had undergone complete resection of stage III melanoma, 5-year RFS rate was 40.8% with ipilimumab versus 30.3% with placebo (HR for recurrence or death, 0.76; P < 0.001) [2]. The OS rate at 5 years was 65.4% in the ipilimumab group compared with 54.4% in the placebo group (HR for death, 0.72; P = 0.001). Although ipilimumab is the only immunotherapy to date to have shown an adjuvant OS benefit, a better RFS benefit has been observed with anti-PD-1 treatment. In a phase III trial of 906 patients who underwent complete resection of stage IIIB–IV melanoma, 1-year RFS rate was 70.5% with nivolumab and 60.8% with ipilimumab (HR for disease recurrence or death, 0.65; P < 0.001) [4]. This RFS benefit was seen in all patient subgroups, including patients with stage IIIB, IIC and IV disease. Improved RFS has also been observed with adjuvant pembrolizumab in a trial of 1019 patients with completely resected melanoma [5]. The patient population in this trial differed slightly from the nivolumab trial in that it included patients with stage IIIA disease but no stage IV patients but results were very consistent with a 1-year RFS rate of 75.4% with pembrolizumab versus 61.0% with placebo (HR for recurrence or death, 0.57; P < 0.001). Thus, we have three prospective phase III randomized controlled trials, each involving around 1000 patients, showing a consistent benefit of adjuvant immunotherapy. Meta-analysis of these data would provide two-times level 1a evidence.

With regard to targeted therapy, a phase III trial of 870 patients with completely resected stage III BRAF-mutated melanoma reported a 3-year RFS rate of 58% with combined dabrafenib plus trametinib versus 39% with placebo (HR for relapse or death, 0.47; P < 0.001) [6]. This RFS benefit was seen across all patient subgroups. There was also a trend towards improved OS in an interim analysis (3-year OS rate of 86% versus 77%), although this did not meet the prespecified interim analysis boundary. However, combined with the immunotherapy studies, these data provide a very strong evidence base in favour of adjuvant treatment.

Recent updates to the American Joint Committee on Cancer (AJCC) staging guidelines highlight the clinical need for adjuvant therapies in melanoma [34]. Sentinel node staging has become mandatory in the most recent AJCC guidelines, which means fewer patients are being diagnosed with stage I/II disease and more with stage III disease [35]. Because of this, we know more patients have micrometastatic disease. These patients cannot be offered neoadjuvant therapy; more routine use of sentinel node biopsy as a staging procedure means fewer patients with palpable nodes and less opportunity for neoadjuvant treatment, which could consequently become somewhat of a niche approach. Moreover, although there are good phase II data to support neoadjuvant therapy, phase III data are lacking. Phase II studies include highly selected patients and have limited duration of follow-up. Neoadjuvant studies have also been associated with worse toxicity than adjuvant, with more patients reporting adverse effects.

In conclusion, the use of adjuvant therapy is supported by multiple, large, well-conducted phase III trials. The adjuvant approach has already shown a survival benefit, has been shown to be effective across all subgroups, which is important given the increase in sentinel node-detected N+ disease, has manageable toxicity, and should be considered the standard of care.

### Hussein A. Tawbi: in favor of neoadjuvant

Patients with stage III melanoma have a 10-year melanoma-specific survival rate of 69%, meaning that almost one-third of these patients will die in this time period. The presence of clinically detected lymph nodes means patients have stage IIIB/C disease and so are candidates for neoadjuvant therapy. If patients have an N1b/N2b or above classification then they have already progressed to stage IIIC disease, with a high 10-year risk of death of 40–75%.

Randomized clinical trials of adjuvant immuno- and targeted therapy have indicated a HR for risk of death of around 0.5, meaning only half of patients benefit. In patients with bulky nodes for whom there is a 70% chance of death within 10 years, a 50% risk reduction would still mean that 35% of patients will die. Thus, the likelihood or recurrence in these stage III disease patients treated with adjuvant therapy remains high. This is illustrated by the COMBI-AD trial, which reported estimated 5-year RFS rates of 54% in the dabrafenib plus trametinib arm versus 37% in the placebo arm [7]. These data indicated that 46% of treated patients still did not achieve RFS while 37% of patients were already cured by surgery alone (i.e. the placebo arm), meaning only 17% of patients benefited from adjuvant treatment. Given this, adjuvant therapy can be considered a rather blunt and somewhat blind instrument, with overtreatment an invariable consequence. Moreover, there are no currently validated biomarkers to identify high-risk patients and no evidence to guide clinicians with regard to the optimal initial treatment option or subsequent risk-benefit analysis during therapy (e.g. if and when to re-challenge patients that experience toxicity). This is despite the involvement of many thousands of patients in clinical trials—many more patients and years of study are required to achieve valid answers to the questions that arise over how best to employ adjuvant therapy.

Locoregionally advanced high-risk melanoma with bulky lymph nodes is truly high risk, with melanoma-specific survival of less than 60%. Neoadjuvant therapy offers the potential to treat more aggressively, especially through the use of various treatment combinations. Neoadjuvant treatment can also help glean insights into how to treat in the adjuvant setting, including the possible use of personalized adjuvant therapy. It involves treating existing disease which is measurable and evaluable both clinically and radiographically. Pathologic response assessment is important and can better delineate the biological impact of treatment. A neoadjuvant approach also means potentially experienced T cells in the tumor microenvironment can be harnessed to help reduce disease burden. High quality and quantity of biospecimens for translational research are provided.

In preclinical experiments, neoadjuvant checkpoint inhibition was superior to adjuvant application in eradicating metastatic disease [36]. In a phase II trial, neoadjuvant plus adjuvant dabrafenib and trametinib significantly improved event-free survival (EFS) versus standard of care (upfront surgery and consideration for adjuvant therapy) in patients with high-risk, surgically resectable, clinical stage III-IV melanoma [18]. This trial was stopped early because of significantly longer EFS in the neoadjuvant plus adjuvant dabrafenib and trametinib arm. PCR rate was 58% in the dabrafenib and trametinib arm and almost no recurrences were observed in patients with a pCR. Correlative studies on longitudinal tumor samples revealed predictors of response and targets of therapeutic resistance, with patients without a pCR having a higher frequency of known resistance-conferring mutations (activating mitogen-activated protein kinases [MAPK]) and immune mechanisms of resistance also being identified (e.g. markers of T cell exhaustion). This illustrates how the neoadjuvant setting can provide a rich source of material to better understand resistance. Similar results have also been seen in a single-arm Australian neoadjuvant dabrafenib plus trametinib study, with a high response rate and high pCR rate in resectable stage III melanoma [37].

In a randomized phase 2 study in 23 patients with high-risk resectable melanoma, treatment with combined ipilimumab and nivolumab yielded high response rates (RECIST ORR 73%, pCR 45%) but substantial toxicity whereas treatment with nivolumab monotherapy yielded modest responses (ORR 25%, pCR 25%) and low toxicity [18]. Tumor samples from this trial revealed known and novel biomarkers and targets for therapeutic resistance, including higher lymphoid infiltrates in responders to both therapies and a more clonal and diverse T cell infiltrate in responders to nivolumab alone.

Similarly, in the OPACIN trial, 20 patients with palpable stage III melanoma were randomized to ipilimumab 3 mg/kg plus nivolumab 1 mg/kg as either four courses after surgery (adjuvant arm) or two courses before surgery and two courses post-surgery (neoadjuvant arm) [19]. Pathological response was achieved in 78% of patients in the neoadjuvant arm with all responders relapse-free after 3-years of follow-up. However, toxicity was high with 90% of patients experiencing grade 3/4 toxicities, making the standard dose used unfeasible. A follow-up trial, OPACIN-NEO, has suggested that neoadjuvant ipilimumab 1 mg/kg plus nivolumab 3 mg/kg has similar response rate but with reduced toxicity and may be appropriate for further investigation [38]. Several other neoadjuvant clinical trials in melanoma are ongoing.

Neoadjuvant treatment also offers clinical, scientific and strategic benefits. From a clinical perspective, it provides more information on patients’ clinical response, allows toxicity assessment in a short time-period and can be used to help guide adjuvant therapy. Scientifically, it allows biomarker development and assessment. Also, in a strategic drug development sense, pharmaceutical companies and regulatory authorities have a favourable outlook towards data from the neoadjuvant setting and this can be used to help accelerate drug development.

To conclude, adjuvant therapy has a role for lower-risk (stage IIIA, some IIIB) and for some intermediate-risk patients (e.g. stage IIB/C) but neoadjuvant therapy is the way forward for patients with clinically detected lymph nodes. The neoadjuvant setting provides great opportunities for rapid advances in our clinical and scientific understanding of new immune- and targeted therapies. However, given the small numbers of patients in studies, there is a need for greater standardization of trial design, endpoints and assessments so that data can be pooled, which has provided the impetus to form the International Neoadjuvant Melanoma Consortium (INMC).

### Key points

For adjuvant therapy:

- Large prospective randomized phase 3 studies.
- More mature data.
- Not only RFS, but also OS benefit.
- Toxicity manageable.
- Works in all subgroups (including SN+ disease).

**Fig. 4:**
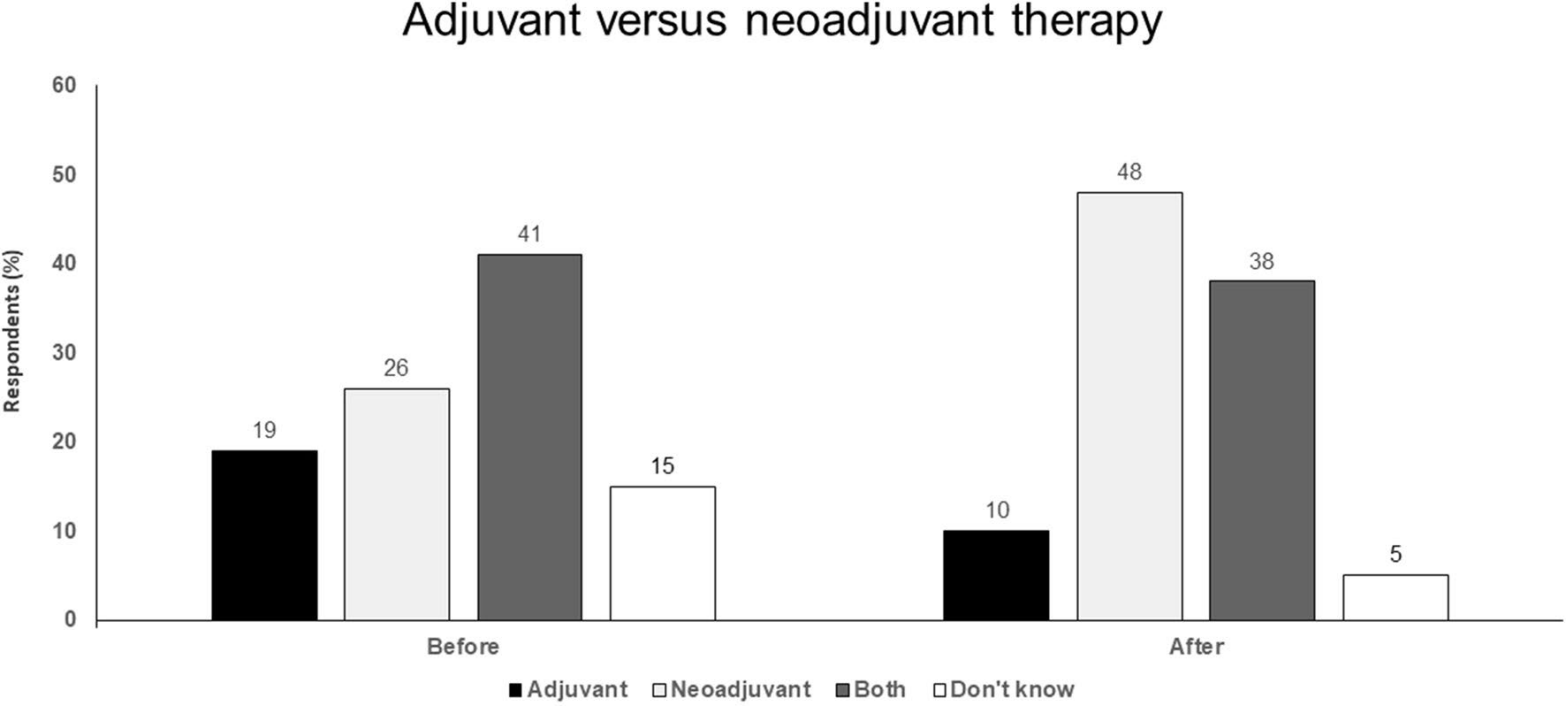
Adjuvant versus neoadjuvant therapy. Audience response before and after debate

For neoadjuvant therapy:

- Bulky regional disease has high risk of relapse.
- Opportunity to use combination therapy and improve surgical outcomes.
- Opportunity to determine therapeutic response and guide adjuvant therapy.
- Pathologic CR associated with excellent outcomes.
- Higher toxicity mitigated by shorter duration (typically 6–8 weeks).
- Opportunity for translational research that offers insights into resistance mechanisms.
- Potential route for registration that could accelerate drug development (Fig. 4).

## Abbreviations

ADCC: antibody-dependent cellular cytotoxicity
AJCC: American Joint Committee on Cancer
CTLA-4: cytotoxic T-lymphocyte-associated antigen
DMFS: distant metastasis-free survival
EFS: event-free survival
EORTC: European Organisation for Research and Treatment of Cancer
FDA: Food and Drug Administration
HR: hazard ratio
IFN: interferon
INMC: International Neoadjuvant Melanoma Consortium
MAPK: mitogen-activated protein kinases
NSCLC: non-small-cell lung cancer
ORR: overall response rate
OS: overall survival
pCR: pathological response rate
PD-1: programmed death-1
PD-L1: programmed death ligand-1
PFS: progression-free survival
RFS: recurrence-free survival
T-Vec: talimogene laherperepvec
TLR: toll-like receptor
Treg: T regulatory cell
